# A Novel Therapy for a Rare Condition: Continuous Anakinra Infusion for a Patient With Macrophage Activation Syndrome

**DOI:** 10.7759/cureus.42968

**Published:** 2023-08-04

**Authors:** Mitch Gullickson, Laura Nichols, Meghan Scheibe

**Affiliations:** 1 Internal Medicine, Sanford Health, Fargo, USA; 2 Internal Medicine, University of North Dakota School of Medicine and Health Sciences, Fargo, USA; 3 Rheumatology, Sanford Health, Fargo, USA

**Keywords:** hemophagocytic lymphohistiocytosis (hlh), adult onset still's disease (aosd), cmv viremia, anakinra, macrophage activation syndrome (mas)

## Abstract

Macrophage activation syndrome (MAS) is a type of hemophagocytic lymphohistiocytosis (HLH), which occurs due to excessive stimulation of the immune system. Common precipitants of MAS include disseminated infection or underlying rheumatologic disorders such as adult-onset Still’s disease which is characterized as an inflammatory arthritis with daily fevers and a salmon-colored rash. We present a case of a patient with probable adult-onset Still’s disease and subsequent disseminated cytomegalovirus (CMV) infection, who met the criteria for MAS based on the presence of a fever, cytopenia in multiple cell lines, elevated ferritin, presence of hemophagocytosis on bone marrow, low fibrinogen, and mild splenomegaly on physical exam. The patient responded to treatment with continuous anakinra infusion and ganciclovir for treatment of CMV. Though cytotoxic medications such as etoposide have traditionally been considered first-line treatment for HLH/MAS, interleukin-1 inhibitors such as anakinra are emerging as aless cytotoxic alternative.

## Introduction

Hemophagocytic lymphocytosis (HLH) associated with underlying autoimmune disease is termed macrophage activation syndrome (MAS) [1]. MAS often carries a poor prognosis, with an all-cause mortality of up to 40% [1]. Early signs and symptoms include purpura, hepatosplenomegaly, altered mental status, cytopenia, and coagulopathy. A cytokine storm ultimately ensues consisting of elevated interferon gamma (IFN-γ), tumor necrosis factor-alpha (TNF-α), soluble CD25, interleukin (IL)-1, and IL-6 [2]. Well-established precipitants of MAS include infection and underlying rheumatologic diseases such as adult-onset Still's disease (AOSD) [1]. AOSD is characterized as inflammatory arthritis similar to the presentation of systemic juvenile idiopathic arthritis, as well as daily fevers and a characteristic salmon-colored rash. 

Current treatment for MAS consists of high-dose glucocorticoid therapy in conjunction with etoposide and/or cyclosporine. Recent case reports and case series such as those by Mehta et al., Griffin et al., and Monteagudo et al. have identified a more targeted IL blockade approach with anakinra as a potential therapeutic option [1-3]. Anakinra is a recombinant IL-1 receptor antagonist that is FDA-approved for the treatment of rheumatoid arthritis at a dose of 100 mg subcutaneously daily. However, this route of administration may be unfavorable in patients with critical illness due to decreased absorption and increased bleeding risk in patients with underlying coagulopathy [1]. The rationale for selecting anakinra over other immunosuppressant agents such as etoposide and cyclosporine is related to the more favorable side effect profile, specifically the decreased risk of hepatoxicity or renal damage [4]. This selective inhibition provided by anakinra serves useful in managing MAS in the setting of active infection: providing a balance between treating the infection and preventing hyperstimulation of the immune system [1].

There are only a small number of published case series and reports discussing the utility of continuous IV anakinra in the management of MAS [1-3]. This case highlights the role of continuous IV anakinra infusion in the setting of macrophage activation syndrome in a patient with suspected AOSD and disseminated cytomegalovirus (CMV) infection and emphasizes the importance of multi-disciplinary care in the management of MAS.

## Case presentation

A 41-year-old female presented with proliferative synovitis in the bilateral wrists, knees, and right ankle. On initial laboratory workup, she was noted to have anemia, elevated acute phase reactants, negative rheumatoid factor, and anti-cyclic citrullinated peptide (anti-CCP) antibodies. The differential diagnosis on initial presentation was broad and included conditions such as seronegative RA, systemic lupus erythematosus (SLE), dermatomyositis, Lyme disease, syphilis, and disseminated gonococcal infection. Antinuclear antibodies (ANA) and other rheumatologic screening antibodies returned negative and infectious disease workup for the aforementioned conditions returned negative as well.

She was diagnosed with probable seronegative RA and started on corticosteroids. Over the course of three months, her symptoms continued to progress. Trials of methotrexate and adalimumab were initiated, and these were unsuccessful as well. Four months following the initial presentation, she developed a bilateral thigh rash concerning for AOSD. Five months following the onset of her symptoms, she presented to the emergency room with worsening of the rash, severe joint pain, and odynophagia. During her admission, she experienced a fever of 101 degrees Fahrenheit, ferritin 4072 ng/mL, aspartate aminotransferase (AST) 413 U/L, fibrinogen 150 mg/dL, and pancytopenia as well as mild hepatosplenomegaly. She underwent upper endoscopy due to odynophagia, which revealed rare viral inclusions on CMV staining; however, CMV viral load was below 35 IU/mL. An infectious disease workup was done for atypical pathogens (Table 1). There was concern for MAS given significantly elevated ferritin in the context of seronegative RA. The patient was started on high-dose steroids and subcutaneous anakinra at a dose of 100 mg three times daily. She was discharged without treatment for CMV, given negligible viral load and improvement in symptoms.

**Table 1:** Infectious Disease Testing Performed During Initial and Subsequent Hospital Admissions EBV: Epstein-Barr virus; CMV: Cytomegalovirus

Test	Result
Infectious disease testing performed during initial admission	
EBV IgG/EBV IgM	Positive/Negative (indicated prior infection)
Parvovirus	Negative
Hepatitis panel	Negative
CMV DNA	Positive (<35 IU/mL)
Influenza A/B	Negative/Negative
HIV 1 Ag/HIV ½ Ab	Negative/Negative
Infectious disease testing performed during subsequent admission	
Leptospira	Negative
Rocky Mountain spotted fever	Negative
*Coxiella burnetii *Q fever	Negative
Bartonella henselae	Negative
Blastomyces	Negative
CMV DNA	Positive (66,500 IU/mL)
Aspergillus	Negative
Coccidoides	Negative
Histoplasma	Negative

Despite initial improvement on this regimen as evidenced by decreasing ferritin levels, stable vital signs, and improvement of odynophagia, the condition of the patient acutely worsened, and she was re-admitted to the hospital for further management. On re-admission, the patient was hypotensive and febrile. Physical examination was notable for an ill-appearing female with jaundice, dry mucous membranes, hepatosplenomegaly, diffuse tenderness to palpation on abdominal examination, and pitting edema of the lower extremities. CMV viral load was 66,500 IU/mL, and ferritin was elevated to >33,500 ng/mL. Infectious disease workup for other atypical pathogens was unremarkable (Table 1). 

**Figure 1:**
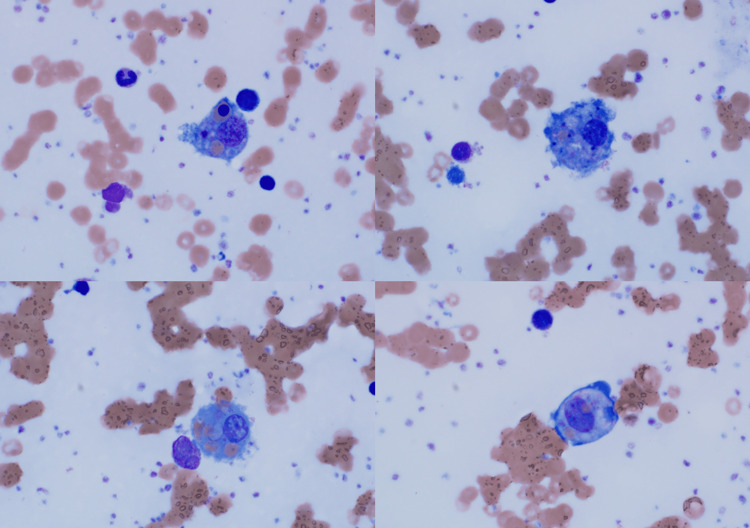
Bone Marrow Biopsy Demonstrating Evidence of Hemophagocytic Histiocytes, 1000X

**Figure 2:**
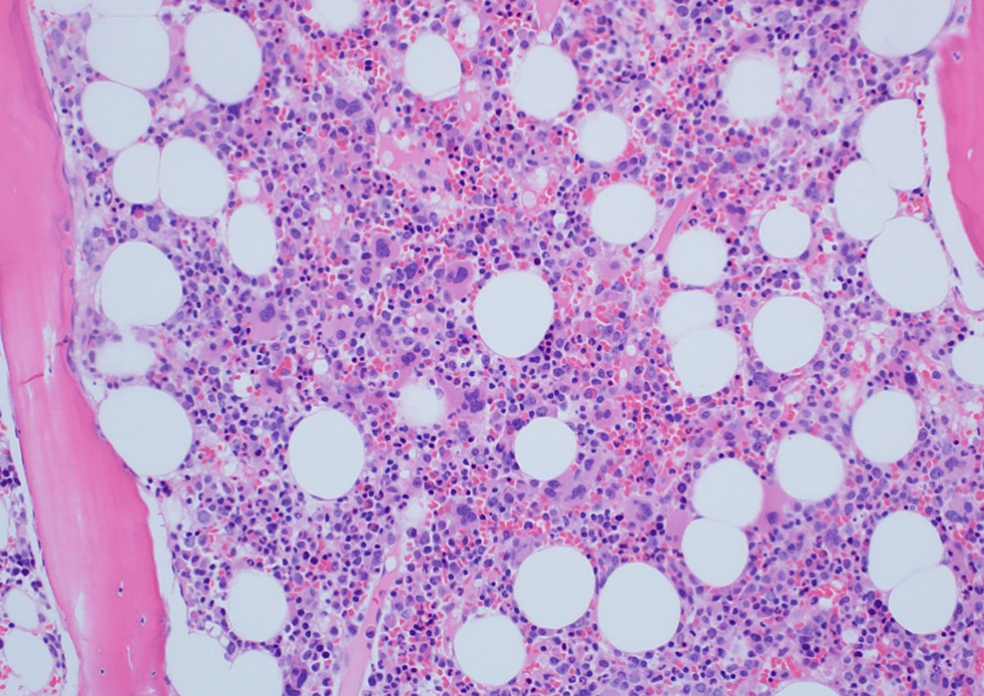
Bone Marrow Biopsy Demonstrating Evidence of Increased Megakaryocytes, H&E 200X

Bone marrow biopsy performed by hematology showed increased hemophagocytic histiocytes and megakaryocytic hyperplasia (Figures 1, 2). The patient met the following criteria for MAS: fever, cytopenia in multiple cell lines, elevated ferritin, presence of hemophagocytosis on bone marrow, low fibrinogen, and mild splenomegaly. She was started on ganciclovir for disseminated CMV and Solu-Medrol with continuous infusion of anakinra, titrated up to a maximum dose of 1.6 mg/kg/hr. The anakinra dose was titrated according to lab parameters, notably ferritin, which served as a surrogate marker for disease progression. Labs were monitored every 12 hours. Over the course of 18 days in the hospital, there was a gradual improvement in ferritin, cytopenia, liver enzymes, and inflammatory markers (Table 2). She was discharged with subcutaneous anakinra and oral valganciclovir. Follow-up with rheumatology was scheduled two days after discharge, and with infectious disease two weeks following discharge.

**Table 2:** Lab Trends Throughout Hospitalization and Associated Anakinra Dosing Infusion dose of anakinra is in mg/kg/hour unless otherwise specified. LDH: lactate dehydrogenase; ALT: alanine transaminase; AST: aspartate aminotransferase

Hospital Day:	Ferritin Level (ng/mL)	LDH (U/L)	Alkaline Phosphatase (U/L)	ALT/AST (U/L)	Total Bilirubin (mg/dL)	Anakinra Dose (mg/kg/hour)
0	>33,500	727	788	562/717	11.6	100 mg daily subcutaneous
1	27,876	509	575	359/196	10.5	1.0
2	10,552	357	519	216/63	10.3	1.0
3	4684	285	559	150/46	9.6	1.5
4	3672	302	642	156/79	11.1	1.5
5	3708	306	692	148/77	10.0	1.6
6	3986	364	768	136/65	8.2	1.6
7	2806	353	832	121/57	6.5	1.6
8	1806	282	771	92/44	4.7	1.5
9	1092	246	695	76/32	4.0	1.25
10	857	238	749	65/31	3.9	1.25
11	683	280	-	-	-	0.75
12	719	298	790	54/31	3.9	0.75
13	694	305	835	57/38	4.1	0.50
14	585	287	824	54/43	4.0	0.33
15	515	281	772	52/45	3.7	500 mg daily subcutaneous
16	505	297	788	52/45	3.7	500 mg daily subcutaneous
17	532	297	854	53/49	3.6	500 mg daily subcutaneous
18	454	-	900	63/65	3.7	500 mg daily subcutaneous

## Discussion

The case presented demonstrates a complex scenario of MAS in a 41-year-old female with suspected AOSD, likely worsened by subsequent immunosuppression-related CMV esophagitis and eventual disseminated CMV infection. Ferritin on admission was >33,500 ng/mL and decreased to 454 ng/mL by the time of discharge, 18 days later. Other diagnoses considered upon hospitalization included bacterial sepsis or ascending cholangitis, which was ruled out by the infectious disease workup and abdominal imaging. The diagnosis of MAS was confirmed with a bone marrow biopsy, which was performed by hematology immediately upon consultation due to the suspicion for MAS.

MAS is often considered the most feared consequence of patients with AOSD and has been reported to occur in close to 15% of these patients [5]. Treatment options for MAS currently include IV Igs, corticosteroids, etoposide, cyclosporine, tocilizumab, and anakinra [6]. Though anakinra is generally administered via the subcutaneous route, the IV route may be preferred in critically ill patients due to increased absorption and a decreased risk of bleeding if an underlying coagulopathy is present [1]. Given the patient’s severely elevated ferritin levels on admission, as well as abnormalities in her platelet count, it was appropriate to transition to an IV administration of anakinra.

Recent case series and reports have highlighted the role of IV anakinra infusion at doses of 1-2 mg/kg in the treatment of patients with macrophage activation syndrome. Monteagudo et al. reported clinical improvement in four out of five of their patients receiving IV anakinra for MAS and reported no adverse effects [3]. Loh et al. reported the usage of IV anakinra in a patient with MAS and AOSD refractory to treatment with methylprednisolone for two weeks, which improved upon initiation of anakinra [7]. There are also many reports of anakinra used to treat MAS in the pediatric literature. Sönmez et al. reported in a cohort of 15 pediatric patients receiving IV anakinra for MAS resulted in 86.6% of patients entering remission following treatment. The only major adverse event reported was the development of vitiligo in one patient [8]. However, despite these positive outcomes, several common point-of-care reference tools continue to cite etoposide and dexamethasone-based therapy as first-line treatment [9,10]. This discrepancy between current practices at academic centers utilizing IL-1 inhibitors and reference materials continuing to recommend etoposide as first-line therapy provided a significant institutional barrier for treating our patient utilizing anakinra. Therefore, continued accumulation of evidence such as the current case report is needed to improve data and determine if traditional first-line therapies should be abandoned in favor of newer, less toxic, and possibly more effective therapies.

Though our patient ultimately did very well with therapy for her MAS and CMV infection, there were several confounding variables that merit further discussion including the definitive diagnosis of both AOSD and MAS. Though the patient met the Yamaguchi criteria for the diagnosis of AOSD with two major (arthralgias lasting two weeks or longer and characteristic rash on the extremities), as well as six minor criteria (sore throat, lymphadenopathy, hepatomegaly, splenomegaly, abnormal liver function tests, and negative ANA and rheumatoid factor) [11], the CMV infection provides a confounding variable regarding the certainty of AOSD diagnosis. The patient’s rash was clearly present prior to the onset of her CMV infection, but other factors such as her hepatosplenomegaly and abnormal liver function tests were noted only when CMV infection may have also been present. Despite these confounders, the patient met the criteria for seronegative RA based on symmetric polyarthritis, negative workup for alternative causes, chronicity greater than six weeks, previously elevated acute phase reactants, and negative CCP and rheumatoid factor. An additional confounder in this case was the level of contribution of CMV to the HLH/MAS presentation. Viral infections such as CMV are known to be a triggering factor for HLH. Regardless of whether the CMV or underlying autoimmune condition was the instigating factor, the treatment for the HLH and/or MAS would remain the same with concomitant treatment of the CMV infection with ganciclovir and treatment of HLH/MAS with IL-1 inhibition. 

## Conclusions

This was a case of a 41-year-old female with an eight-month history of suspected AOSD who presented to the emergency department with severe odynophagia, joint pain, and fever. Lab results on admission were significant for cytopenias in multiple cell lines and ferritin >33,500 ng/mL. Infectious disease workup revealed disseminated CMV infection. Given the suspected underlying rheumatologic disease and concern for MAS, rheumatology and hematology were consulted immediately, and the patient was started on continuous anakinra infusion along with ganciclovir for treatment of CMV. After an 18-day hospital course, the patient’s lab parameters and clinical condition improved. She was deemed stable for discharge with close follow-up with rheumatology and infectious disease. This case provides further support for the role of selective IL-1 inhibition in the treatment of MAS via continuous infusion of anakinra. Additionally, it emphasizes the importance of early recognition and intervention in the management of MAS. Consultation and collaboration with rheumatology, infectious disease, and hematology allowed for the provision of high-quality, patient-centered care.

## References

[1] Mehta P, Cron RQ, Hartwell J, Manson JJ, Tattersall RS (2020). Silencing the cytokine storm: the use of intravenous anakinra in haemophagocytic lymphohistiocytosis or macrophage activation syndrome. Lancet Rheumatol.

[2] Griffin G, Shenoi S, Hughes GC (2020). Hemophagocytic lymphohistiocytosis: an update on pathogenesis, diagnosis, and therapy. Best Pract Res Clin Rheumatol.

[3] Monteagudo LA, Boothby A, Gertner E (2020). Continuous intravenous anakinra infusion to calm the cytokine storm in macrophage activation syndrome. ACR Open Rheumatol.

[4] Mehta P, McAuley DF, Brown M, Sanchez E, Tattersall RS, Manson JJ (2020). COVID-19: consider cytokine storm syndromes and immunosuppression. Lancet.

[5] Giacomelli R, Ruscitti P, Shoenfeld Y (2018). A comprehensive review on adult onset Still's disease. J Autoimmun.

[6] Carter SJ, Tattersall RS, Ramanan AV (2019). Macrophage activation syndrome in adults: recent advances in pathophysiology, diagnosis and treatment. Rheumatology (Oxford).

[7] Loh NK, Lucas M, Fernandez S, Prentice D (2012). Successful treatment of macrophage activation syndrome complicating adult Still disease with anakinra. Intern Med J.

[8] Sönmez HE, Demir S, Bilginer Y, Özen S (2018). Anakinra treatment in macrophage activation syndrome: a single center experience and systemic review of literature. Clin Rheumatol.

[9] (2023). Hemophagocytic Lymphohistiocytosis (HLH) in Adults. https://www.dynamed.com/condition/hemophagocytic-lymphohistiocytosis-hlh-in-adults.

[10] McClain K (2022). Treatment and prognosis of hemophagocytic lymphohistiocytosis. UpToDate.

[11] Macovei LA, Burlui A, Bratoiu I (2022). Adult-onset Still's disease-a complex disease, a challenging treatment. Int J Mol Sci.

